# Contrasting offshore and onshore strong ground motions from the 2025 Mw 7.6 Aomori subduction interface earthquake: Characteristics and model evaluation

**DOI:** 10.1371/journal.pone.0354746

**Published:** 2026-10-14

**Authors:** Mingji Liu, Zhenyu Qu, Haoyu Rong, Lili Kang

**Affiliations:** 1 Department of Architectural Engineering, Shandong Urban Construction Vocational College, Jinan, China; 2 Key Laboratory of Earthquake Engineering and Engineering Vibration, Institute of Engineering Mechanics, China Earthquake Administration, Harbin, China; 3 Key Laboratory of Earthquake Disaster Mitigation, Ministry of Emergency Management, Harbin, China; Henan Polytechnic University, CHINA

## Abstract

On December 8, 2025, an Mw 7.6 subduction interface earthquake occurred off the coast of Aomori Prefecture, Japan, representing the largest-magnitude event recorded to date by the Seafloor Observation Network for Earthquakes and Tsunamis (S-net). Using a dataset comprising 84 offshore and 418 onshore strong-motion records, this study systematically compares the spatial distributions and attenuation characteristics of peak ground acceleration (PGA), spectral accelerations across multiple periods, and significant duration metrics (D70 and D90). The predictive performance of existing onshore and offshore ground-motion models (GMMs) is quantitatively evaluated. Our key findings are as follows: (1) Offshore ground motions exhibit markedly slower attenuation and systematically longer durations than their onshore counterparts. The region of maximum PGA is displaced from the epicenter and concentrated in areas with unburied seafloor stations, whereas the spatial centroid of long-period (T = 5.0 s) spectral acceleration shifts back toward the source region. This pattern indicates that short-to-intermediate-period components are predominantly controlled by local sedimentary layers, while long-period components are governed by source processes. (2) Observed ground motions from this event are generally lower than median predictions from existing GMMs, although most observations fall within ±1 standard deviation of the predictions. Offshore-specific models outperform onshore models in predicting offshore PGA and short-to-intermediate-period spectral accelerations, while onshore duration models systematically underestimate offshore significant duration. (3) Intra-event residual analysis reveals that sediment thickness (ST) estimated from seismic reflection surveys serves as a more spatially stable site-characterization parameter than D1400 (depth to shear-wave velocity of 1.4 km/s). Moreover, water depth exhibits a nonlinear influence on prediction residuals, suggesting that a piecewise functional representation may be more appropriate. Collectively, these results underscore the necessity of employing region-specific ground-motion input models that distinguish between offshore and onshore conditions in marine seismic design and offshore hazard assessments, and highlight the importance of developing independent offshore duration prediction models.

## Introduction

At 23:15 local time on December 8, 2025, a powerful earthquake with a moment magnitude (Mw) of 7.6 struck off the coast of Aomori Prefecture, Japan. The hypocenter was located at 41.0°N, 142.3°E, at a depth of approximately 50 km. Focal mechanism solutions indicate a low-angle thrust event occurring along the active subduction interface of the Pacific Plate. Importantly, this earthquake was comprehensively recorded by the Seafloor Observation Network for Earthquakes and Tsunamis (S-net), marking the largest-magnitude event captured since the network's inception. This event thus provides a unique and valuable dataset for investigating the strong ground motion characteristics of major subduction zone earthquakes.

Offshore ground motions differ fundamentally from onshore motions in several respects, including source properties, propagation path effects (e.g., transmission through oceanic crust and seawater), and local site conditions (e.g., soft seafloor sedimentary layers) [1–6]. The direct application of ground-motion models (GMMs) developed exclusively from onshore records may lead to significant underestimation of offshore ground-motion intensities, thereby posing potential risks to critical marine infrastructure such as submarine pipelines and offshore platforms [7,8]. With the establishment of seafloor observation networks like S-net, considerable progress has been made in characterizing offshore ground motions. For instance, Dhakal et al. [3] analyzed S-net records and found that for periods exceeding 0.5 s, offshore spectral accelerations are substantially larger than onshore values, with differences observed between stations in shallow and deep water. Cui et al. [9] examined the 2021 Fukushima subduction intraslab earthquake and reported that offshore peak ground velocities and long-period spectral accelerations significantly exceed those on land, accompanied by a shift of the dynamic amplification factor spectrum toward longer periods. A comparative study by Tan and Hu [10] further revealed that unburied seafloor stations record higher ground-motion intensities than buried stations, and that offshore ground motions attenuate more slowly and exhibit longer durations.

Leveraging these growing observational datasets, several offshore-specific GMMs have been developed. Hu et al. [11] proposed a model for the Sagami Bay region, demonstrating that soft seafloor sediments substantially amplify intermediate- and long-period motions. Hu et al. [12] subsequently developed an offshore model for the Japan Trench area that distinguishes among different source types (interface, intraslab, shallow crustal, and upper mantle). To better capture spatial variability, Hu et al. [13] and Liu and Hu [14] introduced non-ergodic offshore models that characterize systematic source, path, and site effects, thereby reducing predictive uncertainty. Tan and Hu [15] developed a horizontal-component model specifically for subduction intraslab earthquakes. Additionally, machine learning techniques have recently been applied to offshore ground-motion modeling [16]. Nevertheless, despite the availability of multiple offshore GMMs based on S-net data, systematic validation studies using a large-magnitude subduction interface event with abundant onshore and offshore recordings remain scarce. In particular, a unified understanding of the spatial evolution of onshore-offshore differences across multiple ground-motion metrics—including PGA, response spectra at various periods, and significant duration—and their underlying physical mechanisms has yet to be fully established.

In addition, numerical, statistical, and data-driven methods have been increasingly used in geoscience and geoengineering problems, including geological reservoir processes, geothermal energy extraction, and wellbore integrity assessment [17–19]. These studies further indicate the broader importance of quantitative modeling in complex subsurface systems, although the present work focuses specifically on empirical offshore strong-motion characterization and GMM evaluation.

In this study, we use strong-motion records from S-net, K-NET, and KiK-net for the 2025 Mw 7.6 Aomori-Oki subduction interface earthquake to conduct a systematic onshore-offshore comparative analysis. First, it provides a unified offshore-onshore comparison of PGA, spectral accelerations at multiple periods, and significant-duration measures for the largest-magnitude interface event ever recorded by S-net; second, it evaluates both offshore-specific and conventional onshore ground motion models (GMMs) using this combined dataset; third, through intra-event residual analysis, it quantitatively identifies systematic biases in existing GMMs and further examines residual trends with respect to seafloor installation condition, water depth, rupture distance, and available site parameters, thereby offering direct evidence for refining offshore ground motion input models.

## Strong motion data and processing

The strong-motion data used in this study were obtained from three observational networks covering Japan, ensuring the availability of high-quality recordings from both offshore and onshore environments. Offshore data were acquired from the Seafloor Observation Network for Earthquakes and Tsunamis (S-net), which provides valuable three-component seafloor acceleration records. S-net stations are classified into two categories based on sensor installation: unburied stations, where the accelerometer is placed directly on the seafloor surface, and buried stations, where the sensor is installed in a pre-excavated trench approximately 1 m deep. These distinct installation conditions may influence the recorded ground-motion characteristics. Onshore data were obtained from the K-NET and KiK-net strong-motion networks, which together constitute a comprehensive dataset for coastal and inland regions in the study area.

To ensure the reliability of the analysis, all raw acceleration records were screened and processed. Records with low signal-to-noise ratios, irreparable spikes, or excessive tilt-induced rotation angles were discarded. For the retained records, a standard processing workflow was applied: baseline correction was performed to remove instrument drift, followed by bandpass filtering using a fourth-order Butterworth non-causal filter with corner frequencies of 0.1 Hz and 30 Hz. This frequency range preserves the engineering band of interest while suppressing high-frequency noise. Detailed descriptions of the data processing procedures can be found in Liu [20] and Tan [21,22]. Following this processing, the final dataset comprises 84 offshore and 418 onshore strong-motion records.

To facilitate a detailed analysis of onshore–offshore differences, the following key parameters were compiled or computed:

(1) Distance metric: Rupture distance (Rrup) was adopted as the primary distance measure. The finite-fault model used for distance calculation was provided by the United States Geological Survey (USGS). In the dataset, rupture distances range from 27.96 to 300 km for offshore records and from 58.32 to 300 km for onshore records.
(2) Site condition parameters: For onshore stations, the time-averaged shear-wave velocity in the upper 30 m (Vs30) was used to characterize local site conditions. For offshore stations, where shallow site information is unavailable, several marine geological and bathymetric parameters were introduced: water depth (directly provided by S-net); D1400, defined as the depth to the layer where shear-wave velocity reaches 1.4 km/s (derived from the deep subsurface model of the Japan Seismic Hazard Information Station, J-SHIS, as estimated by Tan and Hu [15]); sediment thickness (ST), based on estimates from seismic reflection surveys by Nishizawa et al. [23]; and seafloor slope, calculated from the high-resolution J-EGG500 bathymetric grid, used to characterize local topographic features.
(3) Seismotectonic classification: Following the earthquake classification scheme for Japan proposed by Zhao et al. [24] and incorporating the USGS Slab 2.0 subduction zone geometry model, the 2025 Aomori Mw 7.6 event is confirmed as a subduction interface earthquake occurring along the Pacific Plate boundary. This classification is essential for selecting appropriate ground-motion models for comparative evaluation. Table 1 provides the basic information of offshore stations and the values of PGA. For the 84 offshore S-net records, 56 records are from unburied stations and 28 records are from buried stations. The two station groups also differ in their bathymetric setting: unburied stations are mainly located in deeper water, whereas buried stations are mostly distributed in shallower water. This classification is used below when interpreting the spatial concentration of high PGA values.

**Table 1:** The basic information and PGA values for offshore stations.

Station	Latitude	Longitude	Water depth (m)	D1400 (m)	Embed type	Rrup(km)	PGA (gal)
S3N23.	38.027	142.574	−1220	1003.2	1	288.57	19.30
S3N24.	38.057	142.234	−849	1048.4	1	287.50	19.49
S3N26.	38.106	141.557	−128	340.8	1	288.52	26.99
S6N10.	39.007	144.592	−6160	1011.5	0	240.51	25.56
S3N25.	38.097	141.896	−230	216.0	1	286.19	17.49
S3N21.	37.971	143.245	−2779	873.6	0	298.18	24.55
S6N11.	38.499	144.454	−6118	1264.4	0	278.55	25.92
S3N15.	38.449	142.838	−1472	1271.7	1	242.11	28.68
S3N22.	37.984	142.907	−1645	974.9	0	293.76	25.24
S3N13.	38.590	142.182	−573	452.6	1	229.55	27.03
S3N19.	38.059	143.744	−5591	1989.7	0	298.01	38.01
S5N11.	41.907	144.696	−2093	1440.4	0	173.58	31.95
S3N16.	38.426	143.170	−2420	1216.8	0	247.53	40.01
S3N12.	38.841	142.182	−558	389.7	1	202.18	34.06
S3N14.	38.499	142.500	−1092	636.1	1	236.73	37.36
S3N10.	38.867	142.821	−1310	855.5	1	196.03	43.20
S5N03.	42.480	145.143	−1985	635.7	0	234.03	44.03
S3N01.	39.450	142.458	−940	537.2	1	133.31	58.57
S4N27.	39.745	142.690	−914	1269.7	1	100.22	51.57
S5N01.	42.769	145.712	−2052	1776.8	0	291.08	41.62
S6N09.	39.517	144.719	−6054	1000.0	0	210.74	66.47
S3N17.	38.398	143.514	−3020	1275.8	0	256.84	42.55
S6N04.	41.665	146.175	−6950	1056.4	0	288.74	55.52
S3N11.	38.935	142.487	−1168	573.9	1	188.94	53.00
S6N07.	40.536	144.938	−6160	1000.2	0	190.61	63.71
S3N18.	38.306	143.781	−5528	1019.0	0	273.07	43.66
S3N04.	39.296	143.454	−2490	1317.9	0	161.89	74.14
S4N28.	39.739	142.341	−569	747.2	1	104.23	73.12
S5N02.	42.640	145.406	−2042	465.8	0	261.94	69.17
S5N04.	42.229	145.204	−3149	987.1	0	225.68	82.81
S5N15.	41.496	144.088	−1950	1425.1	0	115.20	102.77
S3N08.	38.783	143.477	−2465	1460.5	0	215.34	80.65
S3N06.	39.046	143.931	−5440	612.7	0	204.20	98.08
S6N08.	40.032	144.809	−5900	1000.0	0	190.88	80.81
S4N23.	39.772	143.926	−4358	980.3	0	141.91	82.15
S5N12.	42.078	144.638	−1772	1211.0	0	176.16	64.60
S3N09.	38.774	143.144	−1779	1437.1	0	209.44	75.57
S4N24.	39.639	143.715	−2936	1136.5	0	140.77	113.24
S5N05.	42.062	145.437	−3813	1050.5	0	237.24	92.13
S3N07.	38.831	143.785	−5231	1321.9	0	219.50	97.14
S5N07.	41.664	145.529	−5953	864.5	0	235.18	103.92
S5N08.	41.530	145.248	−5980	1263.4	0	210.52	122.34
S5N13.	41.979	144.345	−1607	3042.6	0	149.66	98.21
S5N10.	41.654	144.672	−4103	713.6	0	164.60	96.42
S4N19.	40.090	142.970	−1256	1345.8	1	68.48	142.37
S4N22.	40.026	143.955	−4107	895.2	0	127.29	129.53
S4N16.	40.330	142.267	−559	434.3	1	50.01	124.40
S4N17.	40.117	142.393	−681	1102.5	1	65.52	119.17
S4N10.	40.433	143.889	−3897	1906.2	0	106.07	194.97
S6N05.	41.372	145.605	−6680	902.2	0	239.90	126.78
S5N14.	41.748	144.178	−1592	2260.3	0	127.72	85.94
S5N09.	41.521	144.905	−5100	609.6	0	182.05	131.29
S3N05.	39.191	143.750	−4308	873.4	0	182.98	127.58
S3N03.	39.323	143.121	−1890	1097.6	0	150.10	196.44
S6N06.	40.900	145.393	−6012	965.5	0	225.63	151.77
S5N06.	41.884	145.654	−5619	929.8	0	249.54	122.61
S3N02.	39.375	142.792	−1622	1223.8	0	140.28	183.63
S4N15.	40.593	142.284	−816	1347.6	1	37.41	251.15
S4N09.	40.552	144.133	−5095	1097.5	0	124.14	159.00
S4N21.	40.086	143.657	−2456	1015.5	0	103.77	214.74
S4N20.	40.074	143.321	−1329	1257.8	1	84.72	144.84
S4N25.	39.698	143.375	−2228	1274.3	0	119.83	204.97
S5N19.	41.561	142.818	−986	2976.7	1	33.15	334.48
S4N18.	40.109	142.622	−969	1411.7	1	62.67	213.13
S4N26.	39.725	143.038	−1569	1280.4	0	106.42	247.57
S4N01.	40.788	141.790	−139	999.2	1	50.39	286.22
S4N02.	40.907	142.106	−858	1410.2	1	42.23	340.63
S5N23.	40.954	141.876	−529	1277.2	1	48.27	368.73
S4N14.	40.593	142.634	−1393	1324.9	1	28.49	253.04
S5N20.	41.610	142.479	−1165	3289.5	1	39.73	319.57
S4N08.	40.781	144.091	−5060	1664.3	0	112.29	261.56
S5N16.	41.374	143.801	−2380	1807.9	0	91.63	435.43
S5N21.	41.425	142.219	−1189	2574.6	1	40.31	410.51
S4N13.	40.520	142.962	−1537	1713.7	0	37.74	625.56
S5N22.	41.199	142.027	−1029	889.2	1	44.65	304.93
S5N18.	41.435	143.126	−1509	1503.1	0	41.73	509.51
S4N12.	40.452	143.202	−1711	2298.5	0	54.44	508.34
S4N11.	40.435	143.543	−2273	2301.5	0	79.41	502.26
S5N17.	41.364	143.453	−2023	899.4	0	64.64	648.61
S4N03.	41.016	142.431	−1425	1719.7	1	33.97	565.05
S4N07.	40.882	143.775	−2613	2740.1	0	93.12	821.65
S4N04.	41.076	142.771	−1798	1685.3	0	27.97	944.22
S4N05.	41.044	143.114	−1940	1999.1	0	42.71	1575.62
S4N06.	40.972	143.448	−1970	2826.2	0	67.06	1129.06

The offshore strong-motion records used in this study were obtained from S-net, and the onshore strong-motion records were obtained from the K-NET and KiK-net networks operated by the National Research Institute for Earth Science and Disaster Resilience (NIED), Japan. The final dataset consists of 84 offshore records from S-net and 418 onshore records from K-NET and KiK-net. Hypocenter information was obtained from the Japan Meteorological Agency (JMA), and the finite-fault/source information used for rupture-distance calculation was obtained from the United States Geological Survey (USGS). Additional site and geophysical parameters were compiled from publicly available datasets, including J-SHIS, Slab2.0, and J-EGG500. All strong-motion records and auxiliary datasets used in this study were obtained from publicly available scientific databases. The collection, processing, and analysis of these data complied with the terms and conditions of the corresponding data providers.

## Spatial distribution of strong motion characteristics

To systematically delineate differences in ground-motion attenuation between offshore and onshore regions for this event, Kriging interpolation was employed to map the spatial distributions of PGA and spectral accelerations at various periods. The results are presented in Fig 1. The Kriging contour maps are used here to illustrate first-order spatial trends rather than exact ground-motion values at unobserved locations. Because S-net stations offshore and K-NET/KiK-net stations onshore have different spatial densities and geometries, the interpolation results may be affected by uneven station distribution and boundary effects, especially in offshore areas.

**Fig 1:**
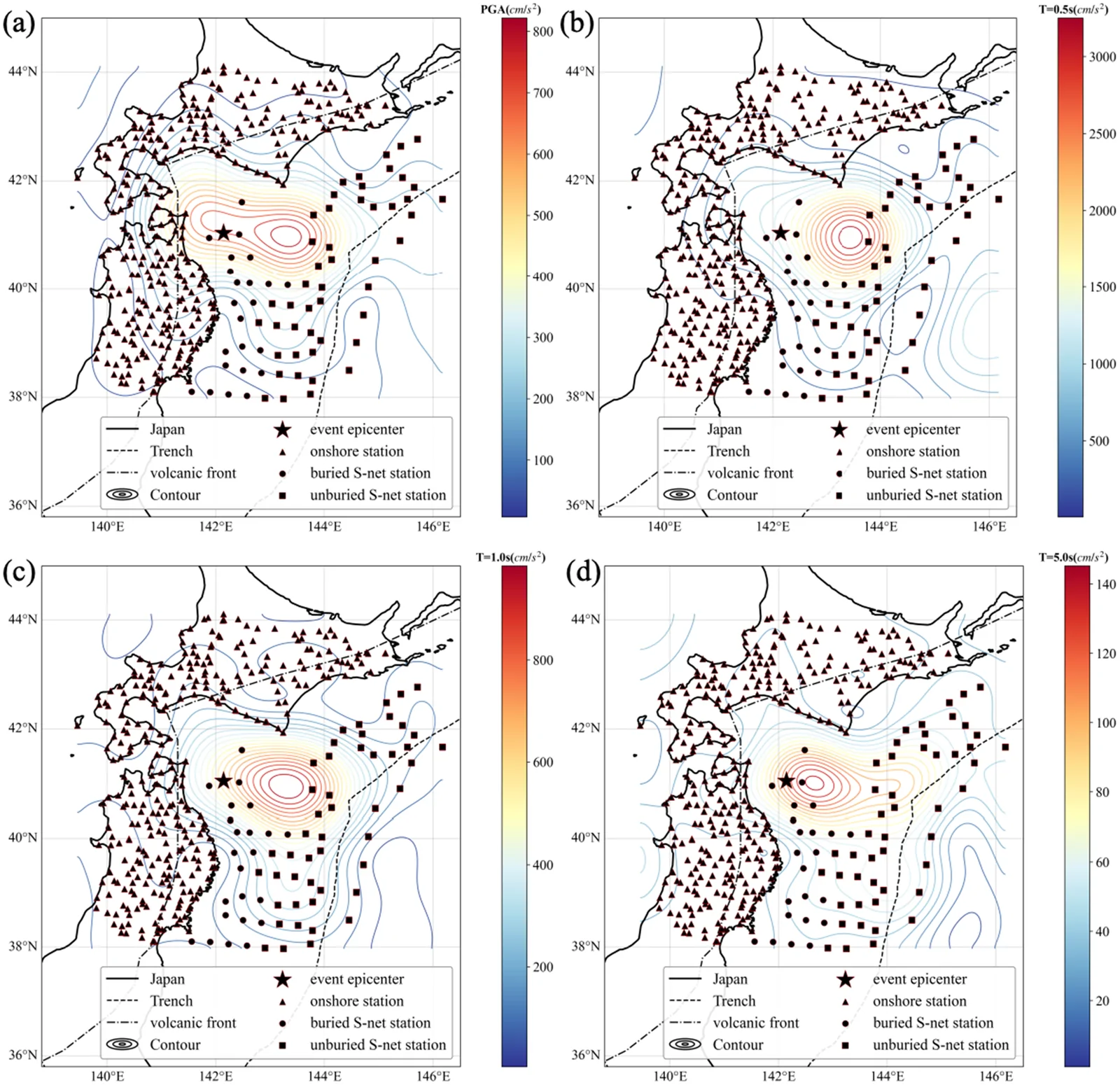
Spatial contours of (a) PGA and spectral accelerations at T = (b) 0.5 s, (c) 1.0 s, and (d) 5.0 s for offshore and onshore ground motions from the 2025 Aomori Mw 7.6 earthquake.

Analysis of Fig 1a reveals that the PGA contours are not symmetrically centered around the epicenter (indicated by a star symbol). Instead, the maximum PGA region is concentrated approximately 80 km from the epicenter, toward the Japan Trench. Notably, all records within this high-PGA area originate from unburied seafloor stations. Previous studies have indicated that unburied stations, owing to direct sensor coupling with seafloor sediments, may be more susceptible to strong nonlinear responses and amplification effects in the surficial layers, yielding significantly higher observed values than those from buried stations and onshore sites [14,15,20,22]. Therefore, differences in station installation conditions likely constitute a key local factor contributing to the anomalous spatial location of the PGA maxima.

As the period increases, the spatial distribution patterns of ground-motion parameters evolve. For short-to-intermediate periods (T = 0.5 s, Fig 1b; T = 1.0 s, Fig 1c), the high-value regions remain approximately centered around the same 80-km-offset area, exhibiting an elliptical shape. This suggests that these components are still jointly controlled by local site effects and possible directivity effects. With further period lengthening, the spatial centroid of the high-value region appears to shift back toward the source area. At long periods (T = 5.0 s, Fig 1d), the maximum spectral acceleration zone is closer to the epicentral area, and the contours elongate approximately parallel to the trench axis. This pattern suggests that long-period motions may be more strongly affected by source rupture processes and regional-scale crustal or subduction-zone structure, although additional source and path analyses are needed to confirm the underlying mechanisms.

A closer comparison of records from unburied and buried stations at comparable distances shows that unburied stations tend to record higher PGA and short-period spectral accelerations. Using PGA ≥ 500 gal as an approximate high-PGA threshold, 10 offshore stations are included in the high-PGA group, of which 9 are unburied stations and 1 is a buried station (Table 2). Thus, unburied stations account for 90.0% of the high-PGA group, and these records are concentrated within Rrup = 27.97–93.12 km. Mann–Whitney rank-sum tests further indicate significant differences between the two station groups at similar distances. For example, within Rrup = 0–100 km, the median PGA is 637.09 gal for unburied stations and 286.22 gal for buried stations, with p = 9.21 × 10^−5^ in Table 3. Within Rrup = 25–120 km, the corresponding medians are 505.30 gal and 253.04 gal, with p = 0.0166. These results support the interpretation that the high-PGA region is dominated by unburied seafloor stations, although source, path, and local site effects may also contribute to the observed spatial pattern.

**Table 2:** Composition of buried/unburied stations within high-PGA ranges.

High-PGA definition	Number of stations	Unburied n	Unburied %	Buried n	Buried %	Rrup min (km)	Rrup max (km)	Rrup median (km)
PGA ≥ 500 gal (approximately top 10%)	10	9	90	1	10	27.97	93.12	48.57
PGA ≥ 300 gal	17	10	58.8	7	41.2	27.97	93.12	42.71
PGA ≥ 250 gal	21	11	52.4	10	47.6	27.97	112.29	42.71

**Table 3:** Similar-distance comparison between unburied and buried stations.

Rrup bin (km)	Unburied n	Unburied median PGA (gal)	Buried n	Buried median PGA (gal)	Median ratio (unburied/buried)	Mann–Whitney U	p-value
0–50	4	784.89	9	334.48	2.35	35	0.0056
50–100	6	578.47	6	143.61	4.03	36	0.0022
100–150	13	159	3	58.57	2.71	39	0.0036
150–200	9	80.81	2	48.1	1.68	16	0.1455
200–300	24	67.82	8	27.01	2.51	172	0.0004
0–100	10	637.09	15	286.22	2.23	146	0.0001
25–120	16	505.3	17	253.04	2	203	0.0166

Fig 2 illustrates the spatial distributions of significant duration metrics, D70 and D90, for this earthquake. The contours exhibit a relatively regular elliptical pattern centered around the epicenter. A comparison of onshore and offshore records shows that, for a given rupture distance, durations recorded at seafloor stations (square symbols) are consistently longer than those at land stations (triangle symbols). This observation provides preliminary evidence that the propagation path through marine media (seawater column and seafloor sedimentary layers) imparts distinct energy dissipation characteristics—manifested as prolonged durations—relative to propagation through terrestrial media.

**Fig 2:**
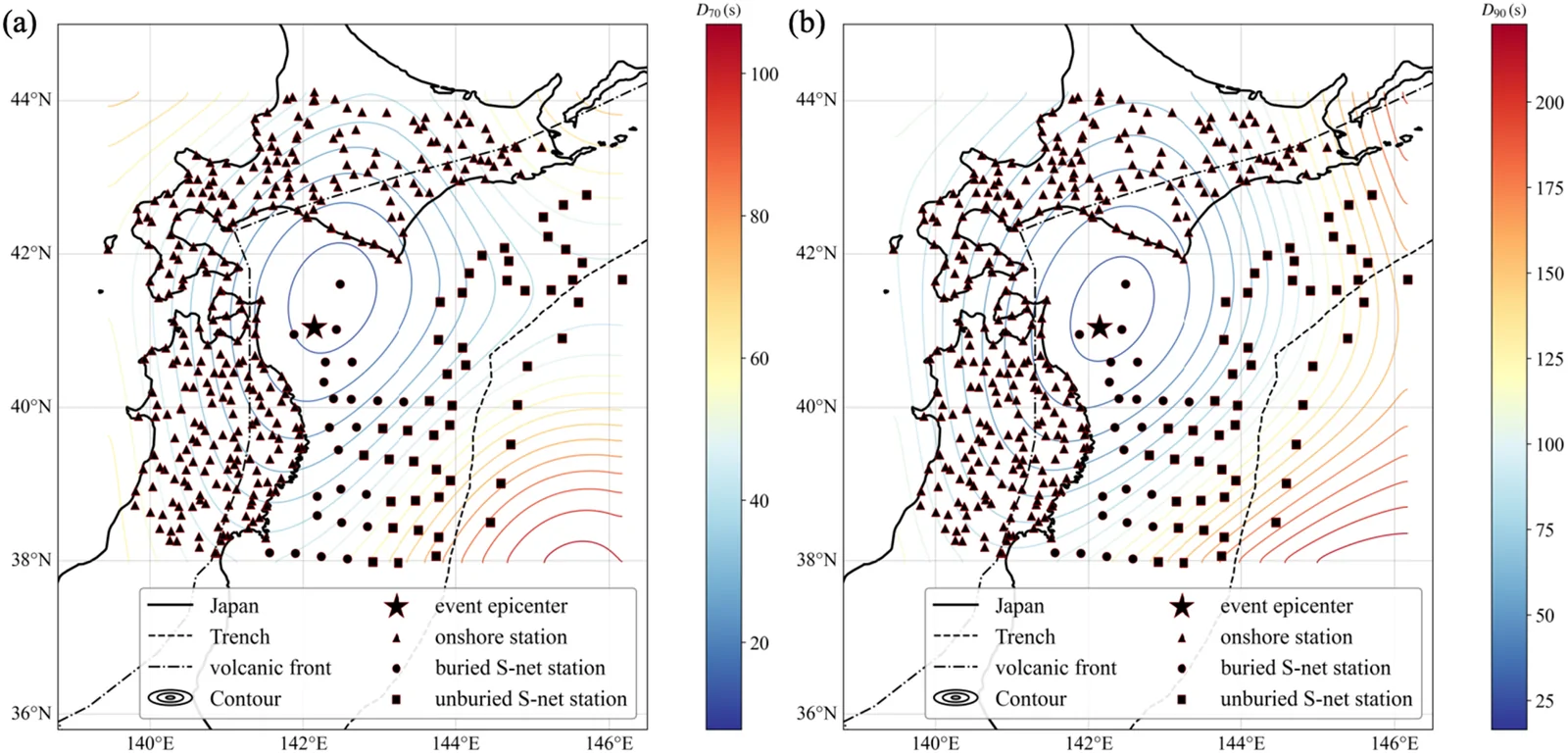
Spatial contours of (a) D70 and (b) D90 significant duration for offshore and onshore ground motions from the 2025 Aomori Mw 7.6 earthquake.

## Characteristics of PGA and response spectra

To quantitatively assess the predictive performance of existing GMMs for this earthquake, we selected and evaluated two representative model classes: (1) onshore GMMs developed for Japanese subduction interface earthquakes (Zhao et al. [25], hereafter Z16; Si et al. [26], hereafter Si2022); and (2) offshore-specific GMMs recently developed using seafloor recordings from Japanese subduction interface events (Tan et al. [27], hereafter Tan2024) and an offshore model derived from an onshore reference empirical approach (Hu et al. [12], hereafter Hu2023INT). This model evaluation aims to address two central questions: (1) Do existing GMMs exhibit systematic prediction biases when applied to the offshore and onshore data from this earthquake? (2) How well do offshore-specific models perform, and what improvements are needed to enhance their applicability and accuracy?

### Evaluation of PGA and spectral acceleration models

Fig 3 compares the observed PGA values for this earthquake with the median predictions of the selected GMMs as a function of rupture distance. The left panel displays offshore data, while the right panel shows onshore data. The solid blue line represents the Tan2024 offshore model, the dashed red line denotes the Hu2023INT model, and the dashed blue lines correspond to various onshore models. The comparison reveals that offshore PGA observations (Fig 3a) attenuate significantly more slowly than onshore observations (Fig 3b). For rupture distances less than ~100 km, the median predictions of onshore models are comparable to the offshore data from buried stations; at larger distances, however, onshore model predictions lie systematically below the offshore observations. This finding reinforces the conclusion that attenuation relationships derived from onshore data cannot be directly extrapolated to accurately represent the unique propagation path and site effects in offshore environments. In contrast, the offshore-specific Tan2024 (solid blue) and Hu2023INT models yield predictions that are generally higher than the observed offshore data from this event. The fact that the observed ground motions are systematically lower helps explain why the reported structural damage and engineering impacts from this Mw 7.6 event were relatively moderate compared to some historical earthquakes of similar magnitude. For the onshore data (Fig 3b), the onshore models exhibit better overall predictive performance, with the Si2022 model showing the closest agreement with the observed onshore attenuation trend, highlighting the adaptability of regional models to local data.

**Fig 3:**
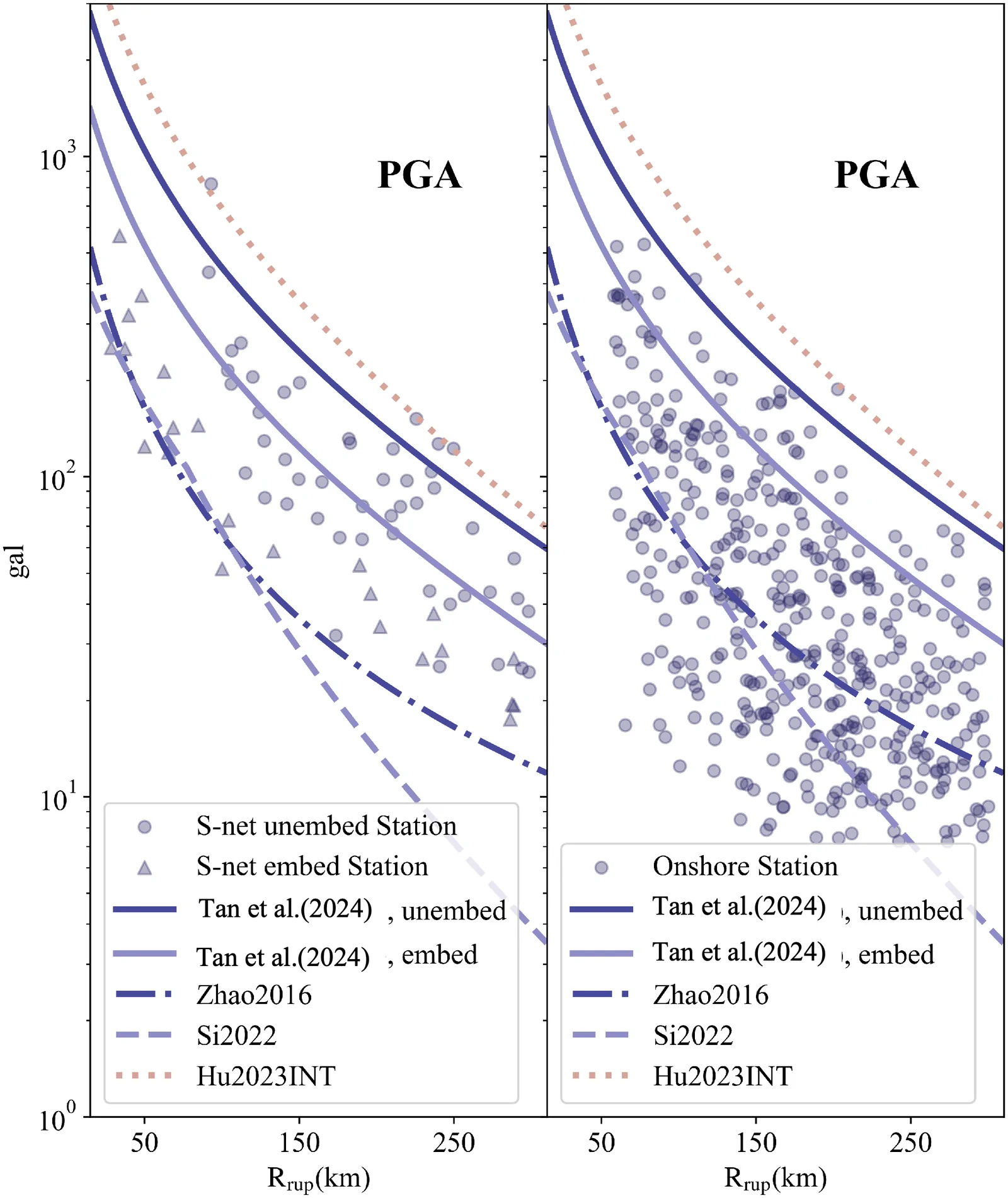
Comparison of observed PGA values with median predictions from selected GMMs for (a) offshore and (b) onshore records in this earthquake.

We further evaluated the predictive performance of the Tan2024 model across multiple spectral periods. Fig 4 presents a comparison of observed offshore spectral accelerations (Sa) at PGA, T = 0.5 s, T = 1.0 s, and T = 5.0 s with the Tan2024 model predictions. At short periods (PGA and T = 0.5 s), the median prediction line of the Tan2024 model passes through the center of the observed data cloud, and the vast majority of data points fall within the ± 1 standard deviation confidence interval, indicating robust predictive capability for high-frequency and intermediate-period components. However, at long periods (T = 5.0 s), the observed data systematically fall below the –1σ lower bound of the model predictions across the entire distance range, revealing a distinct and systematic overestimation of long-period components by the model. This discrepancy suggests that for this Mw 7.6 event, the long-period ground-motion energy may be less pronounced than the typical predictions of current offshore models.

**Fig 4:**
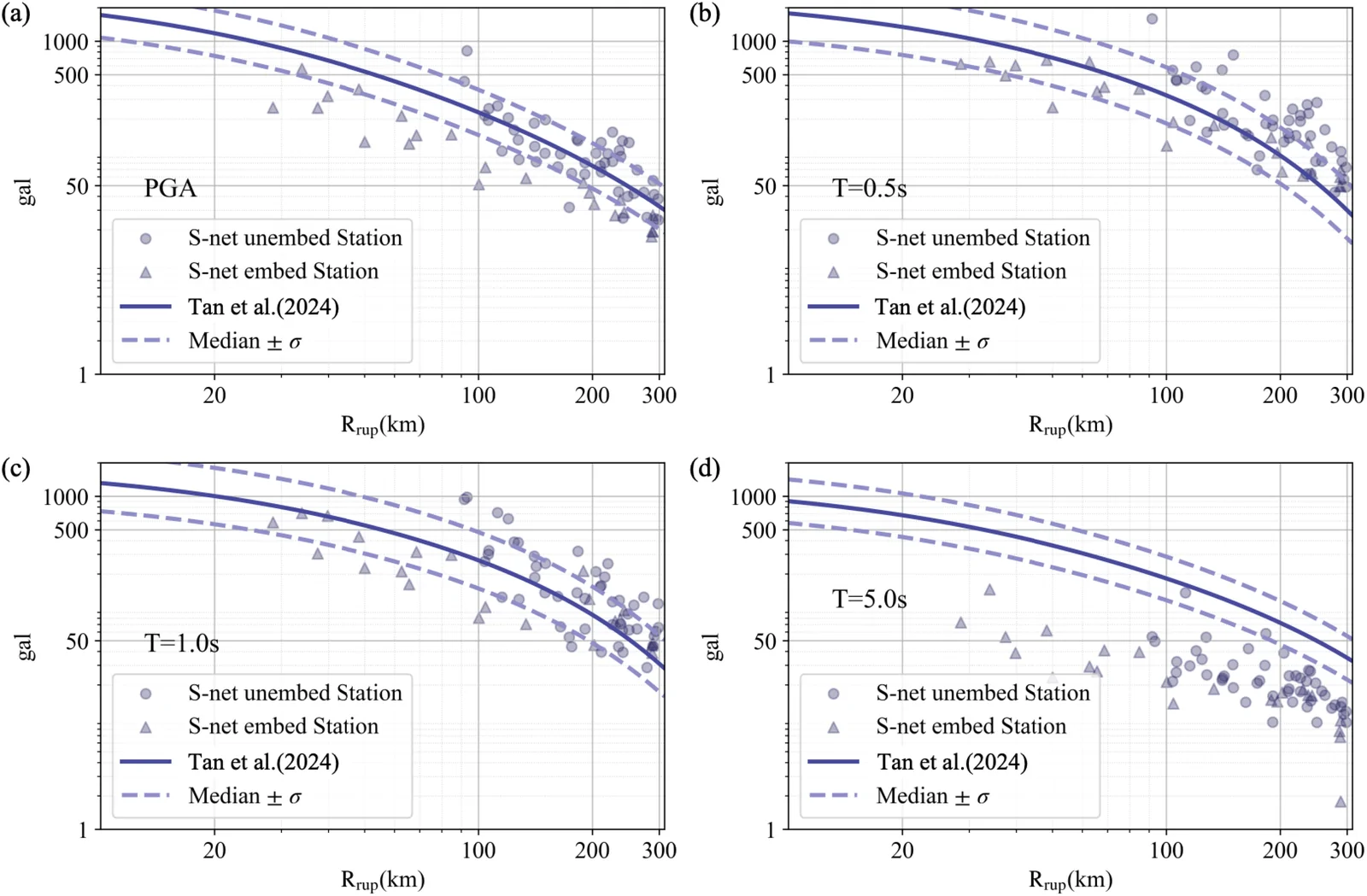
Comparison of offshore observations with predictions from the Tan2024 model at (a) PGA, (b) T = 0.5 s, (c) T = 1.0 s, and (d) T = 5.0 s.

The systematic overestimation at T = 5.0 s may be related to event-specific source and path effects. Possible explanations include relatively weak long-period energy release for this particular interface event, rupture directivity that did not favor the offshore stations at long periods, and long-period path effects associated with the regional subduction-zone structure. This discrepancy does not necessarily invalidate the Tan2024 model, because many observations remain within the model uncertainty range; however, it indicates that long-period predictions for large interface earthquakes may exhibit stronger event-to-event variability than PGA and short-to-intermediate-period spectral accelerations.

For reference, Fig 5 shows the analogous comparison for onshore observations and the Zhao2016 model at the same periods. The median predictions of the Zhao2016 model align well with the central trend of the onshore observations, and most data points lie within the model's confidence intervals. This confirms that the onshore ground-motion characteristics of this earthquake fall within the reasonable predictive range of existing onshore models, thereby underscoring the unique nature of offshore ground motions and the associated modeling challenges.

**Fig 5:**
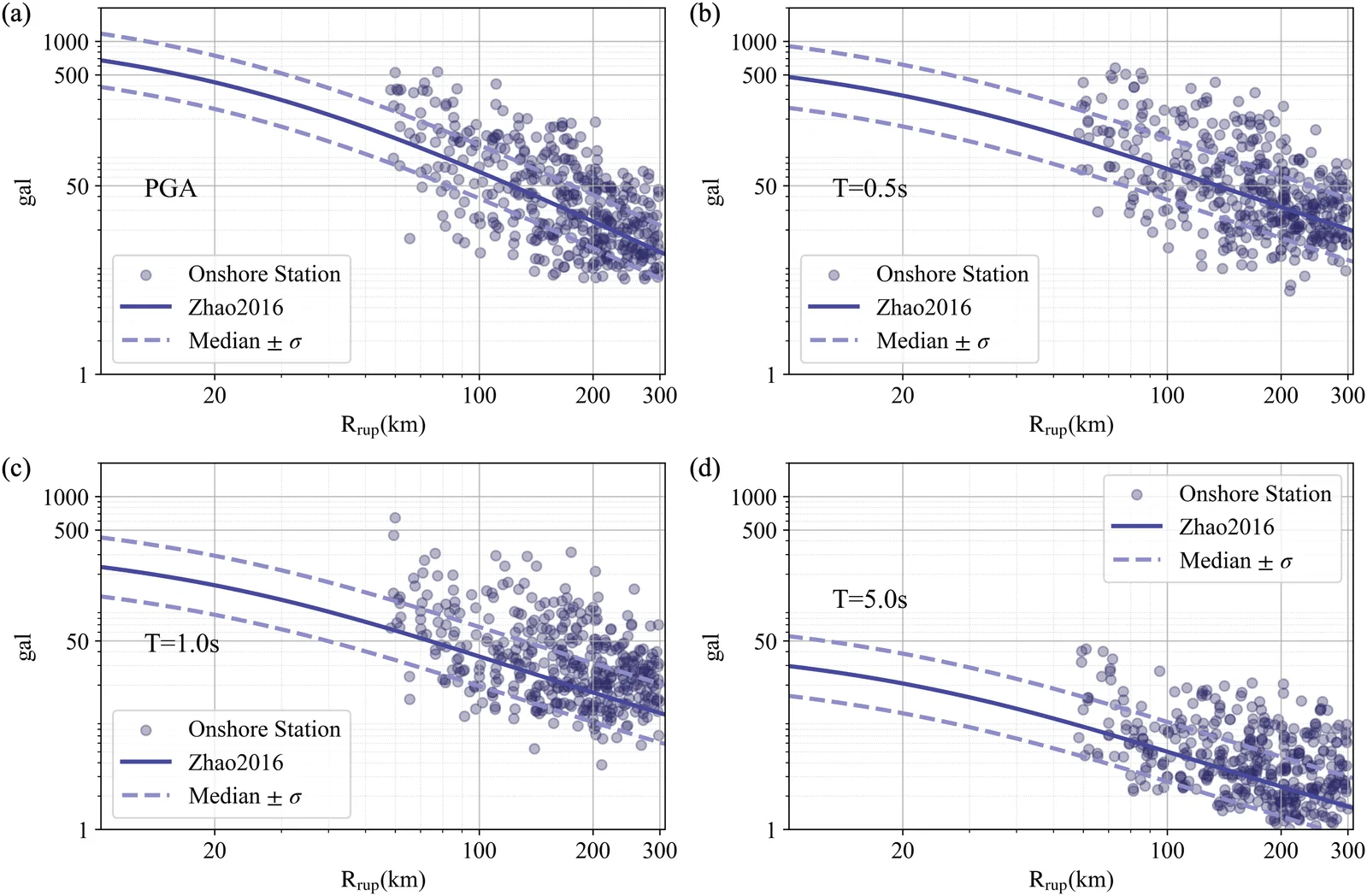
Comparison of onshore observations with predictions from the Zhao2016a model at (a) PGA, (b) T = 0.5 s, (c) T = 1.0 s, and (d) T = 5.0 s.

### Intra-event residual analysis

In the preceding model evaluation, parameters other than distance were set to dataset average values when computing predictions. This approach may introduce bias due to non-uniform parameter distributions. To more accurately assess systematic model errors, we computed intra-event residuals—defined as the logarithmic difference between observed and predicted values at each station. We focus on the relationship between residuals from the Tan2024 model and key seafloor site parameters to diagnose sources of error and identify avenues for model improvement.

1. **Influence of Sediment Thickness Characterization Parameters**

The Tan2024 model employs D1400 (depth to Vs = 1.4 km/s) as the primary parameter representing sediment thickness. Fig 6 compares the distribution of residuals with respect to D1400 and with respect to sediment thickness (ST) estimated from seismic reflection surveys by Nishizawa et al. [23] (note: some stations lack ST values and are plotted as zero). In this event, the residual patterns do not show a strong systematic dependence on either D1400 or ST. Therefore, sediment thickness, at least as represented by the available D1400 and ST indicators, appears to be a secondary factor for the intra-event residual pattern.

**Fig 6:**
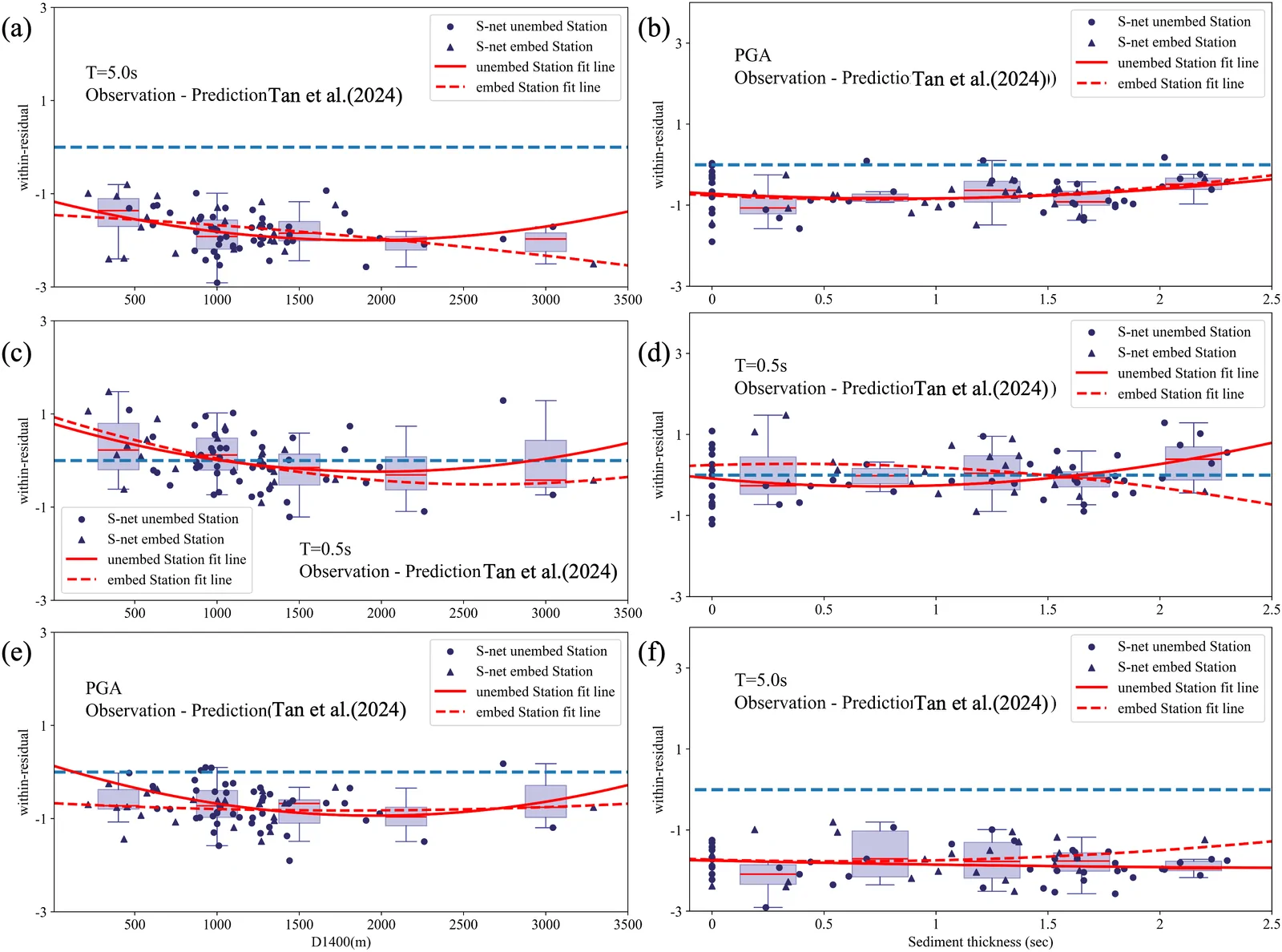
Distribution of intra-event residuals for offshore data versus (a, c, e) D1400 and (b, d, f) ST.

2. **Influence of Water Depth and Seafloor Topography Parameters**

Fig 7 further illustrates the distribution of intra-event residuals with respect to water depth and seafloor slope. For water depth (Fig 7a, c, e), the overall mean residual is negative except at T = 0.5 s, indicating a tendency of model overestimation for this event. The residuals do not show a strong monotonic correlation with water depth; however, binned residual means suggest possible changes around water depths of approximately 1500 m and 4500 m. Therefore, the influence of water depth is described as a possible nonlinear or depth-dependent trend rather than as a statistically confirmed monotonic effect. Regarding seafloor slope (Fig 7b, d, f), the correlation with intra-event residuals remains weak, suggesting that local seafloor slope is not a dominant parameter controlling prediction errors for this event.

**Fig 7:**
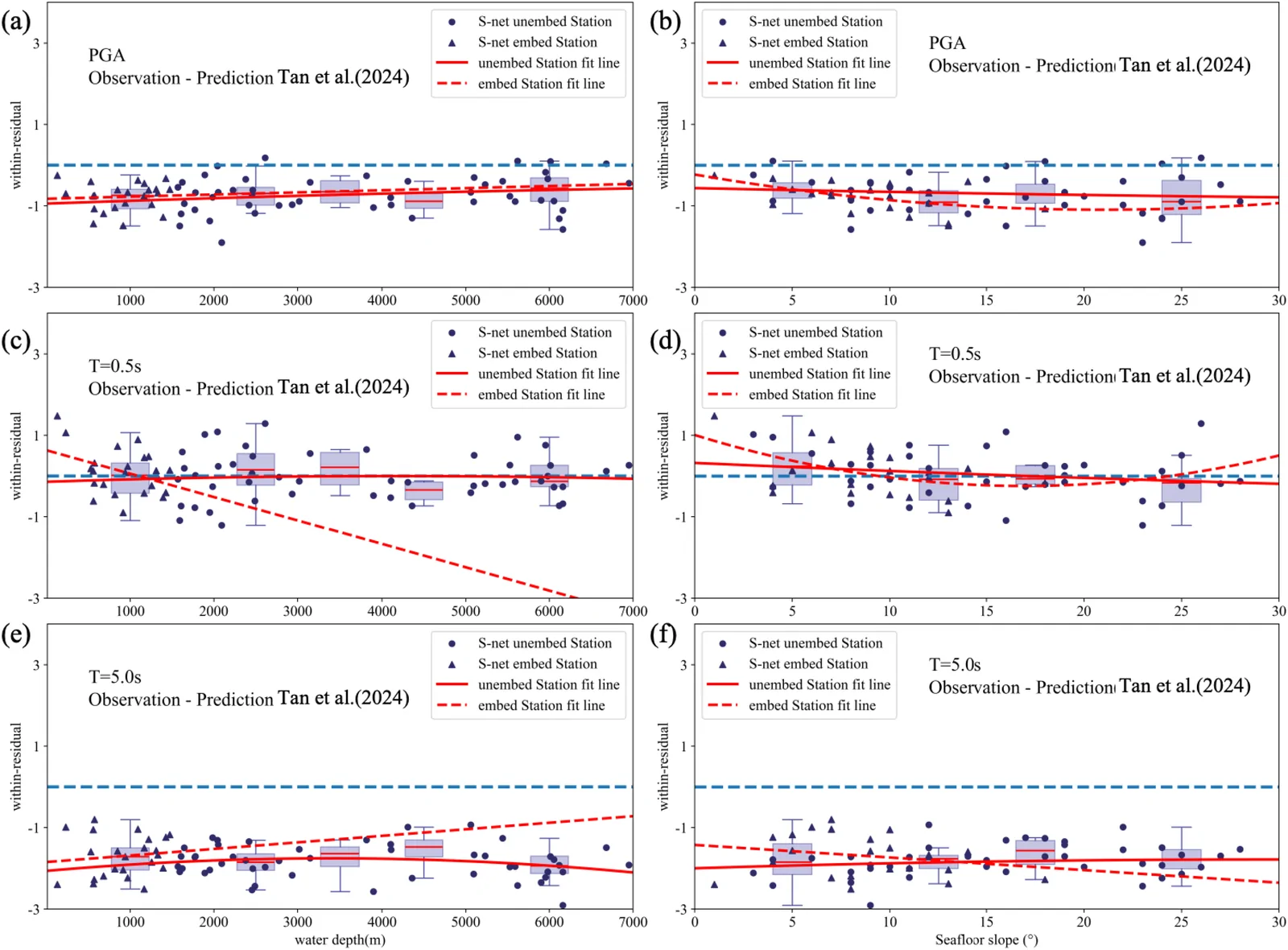
Distribution of intra-event residuals for offshore data versus (a, c, e) water depth and (b, d, f) seafloor slope.

## Significant duration characteristics

To evaluate the predictive capability of existing models for ground-motion duration in this earthquake, we selected the duration prediction model developed by Zhao et al. [28] (hereafter ZGTZ23) as a benchmark. This model, derived from onshore records of subduction interface and intraslab earthquakes in Japan, incorporates the effects of magnitude, distance, and site conditions and has demonstrated good applicability for onshore ground-motion analysis in Japan.

Fig 8 compares observed significant durations (D70 and D90) from this earthquake with predictions from the ZGTZ23 model. Overall, onshore durations (Fig 8b, d) agree well with model predictions, with most data points falling within the ± 1 standard deviation bounds. This indicates that existing onshore duration models provide reliable predictions for the onshore ground motions in the study region. In contrast, offshore durations (Fig 8a, c) exhibit markedly different behavior: across the entire distance range, observed offshore durations are systematically and substantially longer than model predictions, and this deviation becomes more pronounced with increasing rupture distance. This comparison further confirms that current duration prediction models developed from onshore recordings exhibit significant biases in characterizing the inherently slower energy dissipation and prolonged durations typical of offshore ground motions.

**Fig 8:**
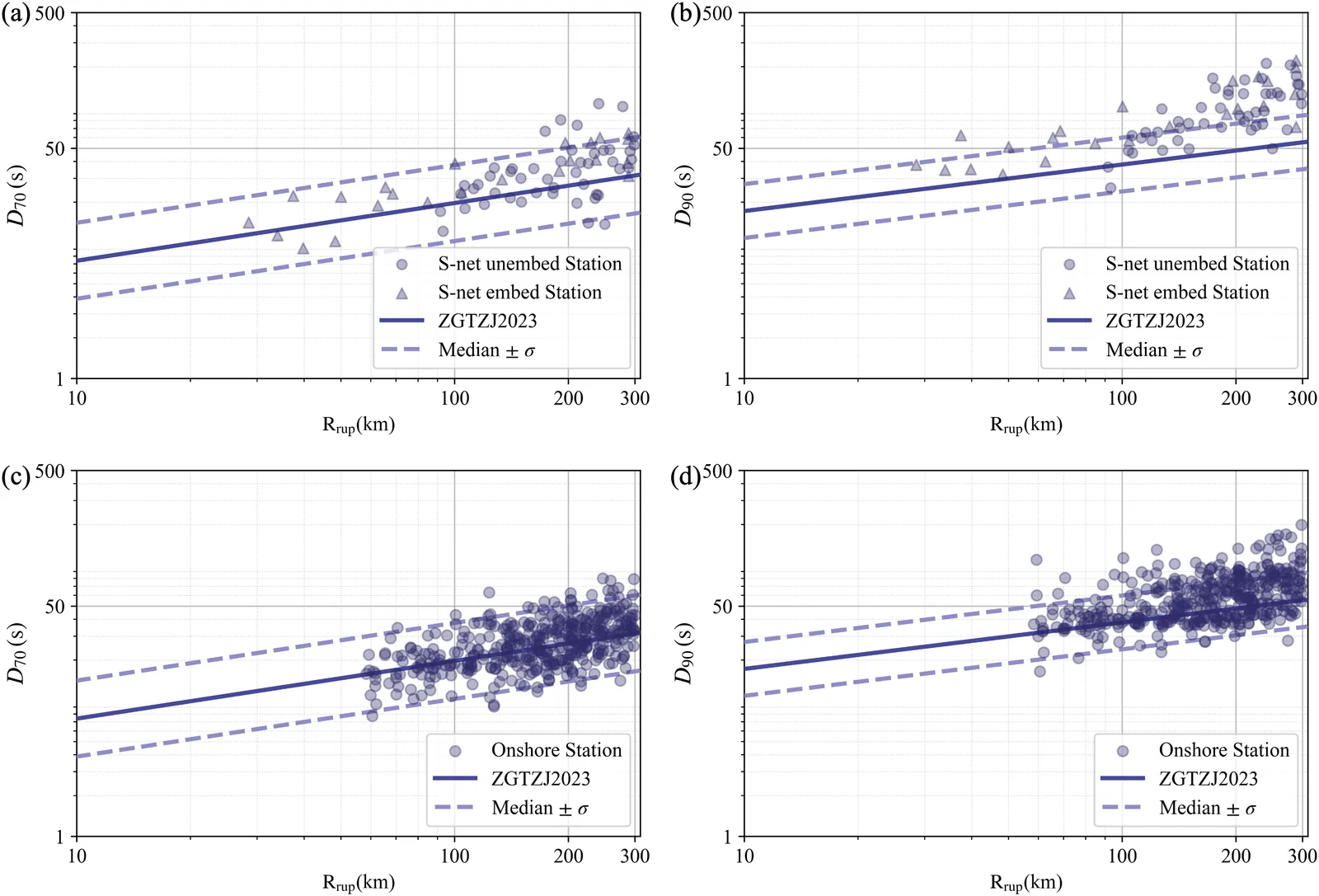
Comparison of observed significant duration versus predictions from the ZGTZ23 model for (a, b) offshore and (c, d) onshore records in this earthquake.

To investigate the physical mechanisms underlying the systematically prolonged offshore durations, we computed intra-event duration residuals and examined their correlations with rupture distance, water depth, and sediment thickness parameters. Fig 9 presents the residual analysis results for the D5-95 duration. The key findings are as follows:

**Fig 9:**
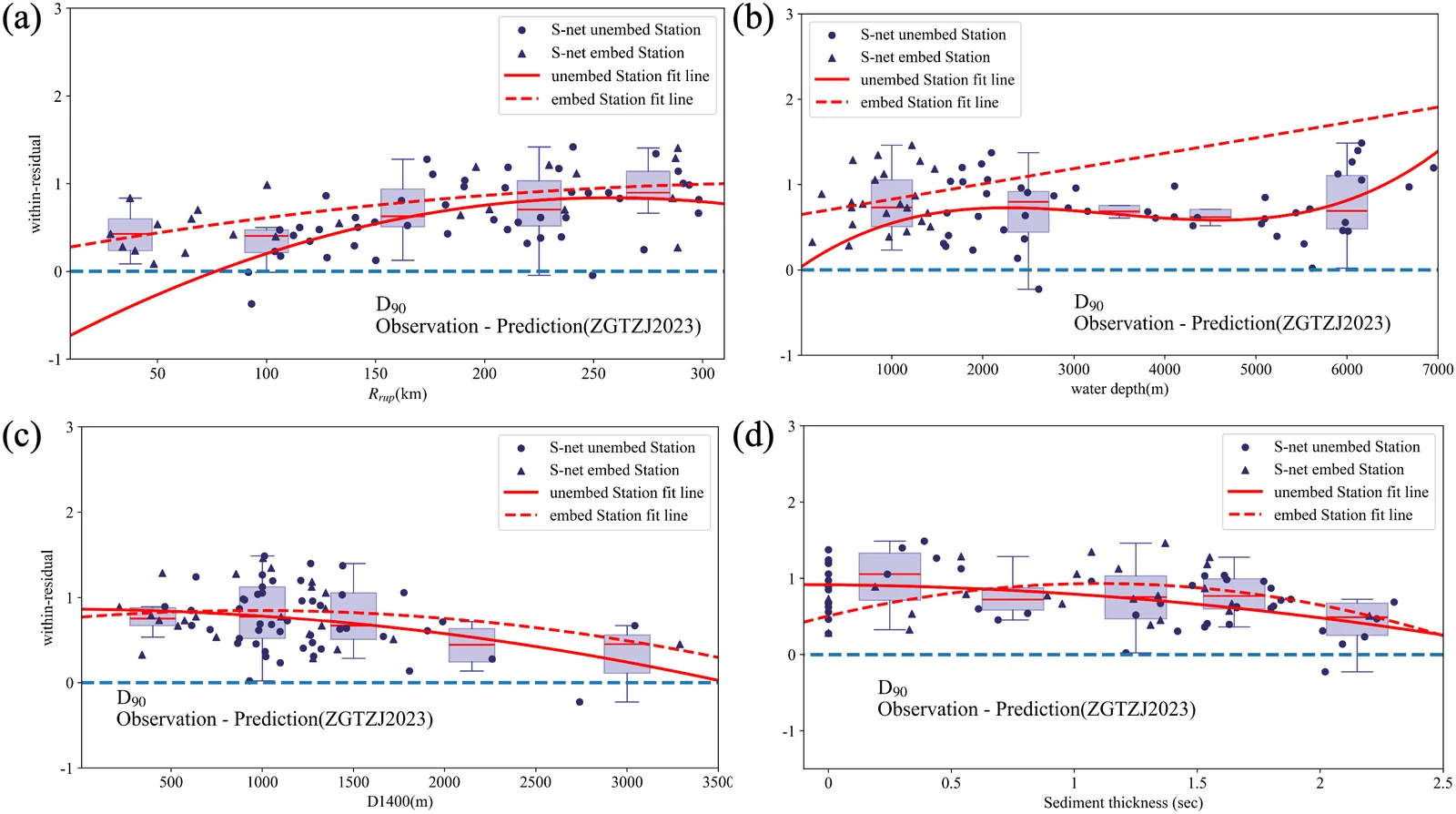
Distribution of intra-event residuals versus (a) rupture distance, (b) water depth, (c) D1400, and (d) ST.

(1) The offshore residuals are predominantly positive, with mean values of 0.375 for D70 and 0.592 for D90, indicating that the ZGTZ23 onshore duration model underestimates offshore significant duration in Table 4. In comparison, the corresponding onshore mean residuals are 0.257 for D70 and 0.243 for D90. Spearman correlation tests show that D90 residuals have a clear positive correlation with rupture distance (ρ = 0.495, p < 0.001), whereas D70 residuals show a weaker but still positive correlation with rupture distance (ρ = 0.231, p = 0.0347).

**Table 4:** Summary of significant-duration residuals based on ZGTZ23 predictions.

Dataset	Residual metric	n	Mean ln(obs/pred)	Median	Std.
Offshore	D70	84	0.375	0.395	0.408
Offshore	D90	84	0.592	0.582	0.414
Onshore	D70	418	0.257	0.264	0.334
Onshore	D90	418	0.243	0.217	0.328

(2) Duration residuals are predominantly positive across the entire distance range, confirming the systematic underestimation of offshore durations by the ZGTZ23 model. Quantitatively, D90 duration residuals increase with rupture distance, with Spearman ρ = 0.495 and p < 0.001. A supplementary regression using ln(Y) = a + b ln(Rrup) also shows that offshore duration has a stronger apparent distance dependence than onshore duration in Table 5. The D70 distance coefficient is 0.6463 for offshore records and 0.5278 for onshore records, while the D90 distance coefficient is 0.6961 for offshore records and 0.4684 for onshore records. These results support the need for offshore-specific duration prediction models.

**Table 5:** Apparent distance-scaling coefficients from supplementary regression.

Metric	Dataset	n	coefficient b	R²	p-value
PGA	Offshore	84	−1.2277	0.6757	9.40 × 10^-22
PGA	Onshore	418	−1.4920	0.3961	1.70 × 10^-47
D70	Offshore	84	0.6463	0.5653	1.69 × 10^-16
D70	Onshore	418	0.5278	0.2899	8.50 × 10^-33
D90	Offshore	84	0.6961	0.6114	3.75 × 10^-18
D90	Onshore	418	0.4684	0.2302	2.00 × 10^-25

(3) The scatter plot of residuals versus water depth (Fig 9b) does not reveal a significant monotonic trend. The Spearman correlation coefficients are ρ = −0.048 with p = 0.6625 for D70 residuals and ρ = 0.053 with p = 0.6332 for D90 residuals. Therefore, water depth alone is unlikely to be the dominant factor responsible for prolonged offshore durations. However, binned residual means suggest possible depth-dependent variation: for D90, the mean residuals are 0.697, 0.464, and 0.698 for water-depth ranges of 0–1500 m, 1500–4500 m, and 4500–8000 m, respectively. This pattern indicates that the relationship may be non-monotonic and should be further examined using larger datasets.
(4) The relationship between duration residuals and sediment-related parameters is relatively weak. For D1400, the D70 residual does not show a statistically significant Spearman correlation (ρ = −0.146, p = 0.1851), whereas the D90 residual shows a weak negative correlation (ρ = −0.327, p = 0.0024). This result suggests that thicker sedimentary structure may slightly reduce D90 residuals, but the effect is secondary compared with the distance dependence. Therefore, the previous conclusion has been revised to avoid describing sediment thickness as a primary controlling factor without sufficient statistical support.

The increase in offshore duration residuals with distance may be associated with two mechanisms. First, scattering, reverberation, and complex wave propagation within the oceanic crust and seafloor sedimentary layers may broaden the energy-arrival window. Second, stronger attenuation of high-frequency components during offshore propagation may stretch the waveform envelope and increase the relative contribution of later-arriving lower-frequency energy. Because the present analysis is empirical, these two mechanisms cannot be fully separated here, and further waveform simulation or path-specific attenuation analysis is needed.

## Conclusions

This study presents a systematic comparative analysis of onshore and offshore strong ground motion characteristics for the December 8, 2025, Mw 7.6 subduction interface earthquake off Aomori, Japan. The analysis is based on a dataset comprising 84 offshore records from S-net and 418 onshore records from K-NET and KiK-net. We evaluated existing GMMs through residual analysis and derived the following principal conclusions:

(1) Within the rupture-distance range covered by the dataset, offshore PGA and short-period spectral accelerations are generally higher than onshore values and show a slower apparent attenuation trend. The apparent distance coefficient of PGA_h is −1.2277 for offshore records and −1.4920 for onshore records based on supplementary regression. The maximum PGA region is displaced from the epicenter and is dominated by unburied seafloor stations; using PGA ≥ 500 gal, 9 of the 10 high-PGA offshore records are from unburied stations. As period increases, the high-value region of spectral acceleration appears to shift back toward the source area, suggesting a stronger contribution of source and regional path effects at long periods.
(2) Onshore records are generally well predicted by existing onshore GMMs. Offshore records, while systematically lower than the median predictions of some offshore models, mostly remain within the model uncertainty range. Offshore-specific models exhibit better predictive consistency for offshore PGA and spectral accelerations at T ≤ 1.0 s than conventional onshore models. However, at T = 5.0 s, the Tan2024 model overestimates the observations for this event, indicating that long-period offshore predictions may be more sensitive to event-specific source spectra, rupture directivity, and path effects.
(3) The residual analysis indicates that D1400 and sediment-related parameters do not provide a strong first-order explanation for the intensity residuals of this event. For duration residuals, D1400 shows no significant correlation with D70 residuals and only a weak negative correlation with D90 residuals. Water depth does not show a significant monotonic correlation with duration residuals, although binned residuals suggest possible depth-dependent variation. These results indicate that offshore site and path effects are complex and may require more flexible parameterization in future offshore GMMs.
(4) Offshore significant durations are systematically longer than onshore durations, and the deviation from the onshore duration model increases with rupture distance. The mean offshore duration residuals are 0.375 for D70 and 0.592 for D90, and D90 residuals have a significant positive correlation with rupture distance (ρ = 0.495, p < 0.001). Supplementary distance-scaling analysis further shows that offshore D70 and D90 increase more strongly with Rrup than onshore durations. These findings support the development of independent offshore duration prediction models.

In summary, this study provides a comprehensive observational analysis of the pronounced differences in attenuation, spectral content, and duration between offshore and onshore ground motions for the 2025 Aomori Mw 7.6 subduction interface earthquake. It validates the applicability and limitations of existing offshore GMMs and offers insights into the parametric representation of water depth and the need for dedicated offshore duration models. These findings have important implications for the seismic design of marine structures, safety assessment of cross-sea infrastructure, and offshore seismic hazard analysis.
